# Isolated Atrial Lead Conduction Delay following Right Atrial Radiofrequency Maze Procedure

**DOI:** 10.5402/2011/475796

**Published:** 2011-06-01

**Authors:** Mackram F. Eleid, Joseph A. Dearani, Win-Kuang Shen

**Affiliations:** ^1^Division of Cardiovascular Diseases, Department of Internal Medicine, Mayo Clinic, 200 First Street SW, Rochester, MN 55905, USA; ^2^Division of Cardiovascular Surgery, Mayo Clinic, 200 First Street SW, Rochester, MN 55905, USA

## Abstract

A 60-year-old man with a dual-chamber implantable cardioverter defibrillator and severe dyspnea on exertion due to apical hypertrophic cardiomyopathy underwent a septal myectomy and radiofrequency maze procedure. Following the procedure a persistent delay in atrial sensing was observed and was most likely a result of iatrogenic conduction delay from right atrial ablation lines. These observations suggest that atrial conduction properties can be altered during the surgical maze procedure and should be considered in the differential diagnosis of sensing or pacing malfunction.

## 1. Case Description

A 60-year-old male with apical variant hypertrophic cardiomyopathy and history of dual-chamber implantable cardioverter defibrillator 18 months before, nonsustained ventricular tachycardia and primary prevention of sudden cardiac death was admitted with New York Heart Association class III dyspnea on exertion that was refractory to medical therapy. He underwent an apical myectomy procedure along with left and right atrial radiofrequency maze procedures [1] for treatment of recurrent atrial fibrillation. 

Prior to surgery, his dual chamber Medtronic Secura D224DRG was interrogated, and the device and lead function were found to be functioning normally. 

The patient underwent successful cardiac surgery. A device interrogation was performed on postoperative day no. 1 while the patient was in atrial fibrillation/flutter, and atrial lead sensing was observed to be normal. For control of the atrial fibrillation, amiodarone 400 mg P.O. twice daily was initiated and metoprolol increased from 50 mg twice daily to 75 mg twice daily.

**Figure 1:**
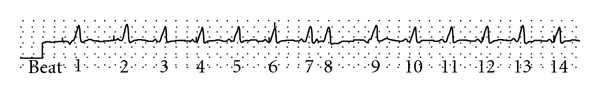


**Figure 2:**
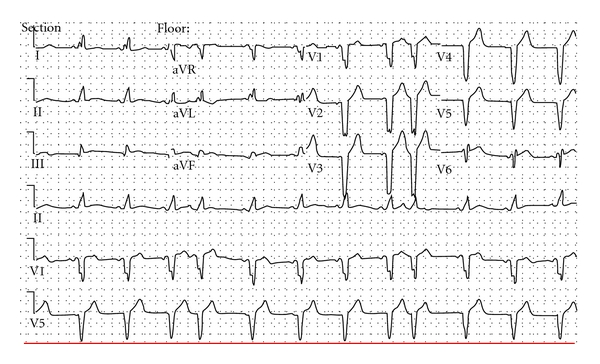


Two days following the surgery, the rhythm converted to sinus, and continuous telemetry revealed the following (Figure 1). The first and ninth beats are paced atrial beats with adequate capture. The pacing spike to the onset of p wave is prolonged (approximately 80 msec) suggesting conduction delay from the pacing site to atrial depolarization. Although a pacing spike is seen prior to the second p wave, due to the pacing site to atrium conduction delay, a native p wave emerges before the paced captured p wave could occur. The fourth beat shows atrial undersensing with an atrial pacing spike after a native p wave. Atrial undersensing is also observed on the 6th, 10th, 12th, and 14th beats. Also note that ventricular safety pacing occurring on beats 4 and 6. 12 lead ECG is shown in Figure 2.

**Figure 3:**
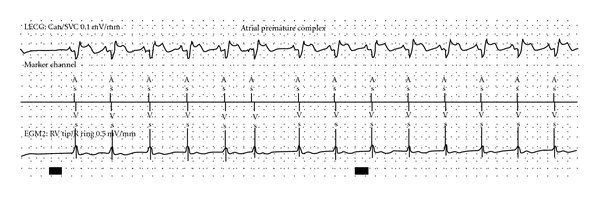


A subsequent device interrogation was performed. Native p waves were sensed late, such that atrial sensing occurred 120 ms after the p wave occurred. p wave amplitude was 3.1 mV (Figure 3). Interestingly, sensing of an atrial premature complex (APC) was different from sinus sensing in that there appeared to be less conduction delay, likely representing an origin with a closer location or more direct conduction pathway to the pacing lead. 

Threshold testing was also performed and found to be normal. A portable chest radiograph was obtained and accounting for differences in technique showed no evidence of atrial lead macrodislodgement compared with preoperative location in the area of the right atrial appendage. 

Because pacing requirements were minimal and ventricular pacing, sensing, and defibrillator thresholds adequate, the device mode was set to VVI at 40 bpm to allow for back-up pacing if needed. 

## 2. Discussion

**Figure 4:**
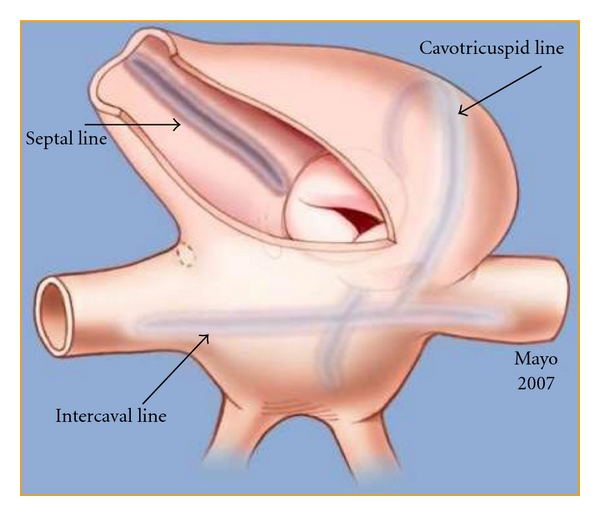
Diagram of right atrial maze ablation lines.

Considering the recent radiofrequency maze procedure performed at the time of cardiac surgery, it was determined that the delayed atrial sensing most likely occurred as a result of surgically created conduction delay from ablation lines [2]. The right atrial maze procedure in this patient consisted of an intercaval line, a septal line, and a cavotricuspid line (Figure 4). Additionally, a horizontal line was made connecting the superior lateral tricuspid annulus with the posterior intercaval line [1]. If a pacing lead is placed on the side of the intercaval line opposite to the sinus node or inferior to the horizontal line, conduction delay from the sinus node to the pacing lead can occur and likely resulted in the observed sensing delay. 

Following the maze procedure, the native p wave typically becomes smaller in amplitude, monophasic and has positive polarity [3]. There is little data in the literature describing the effects on the maze procedure on atrial pacing and sensing function. To the best of our knowledge, this is the first report of sensing delay occurring after a surgical maze procedure. In the present case, undersensing during sinus rhythm was most likely due to intermittent intra-atrial conduction block to the pacing tip [4]. The atrial premature complex associated with earlier sensing in comparison with the sinus rhythm could be explained by a more direct conduction pathway not encountering the linear line of block or delay [5]. These observations highlight the alteration of atrial conduction properties that occurs following the surgical maze procedure and should be considered in the differential diagnosis of pacemaker sensing or pacing malfunction. This occurrence has the potential to result in pacemaker system malfunction which may necessitate lead revision in some patients. Attempts to avoid lead isolation by ablation lines may potentially help to prevent this complication.

## References

[1] Sie HT, Beukema WP, Misier AR (2001). Radiofrequency modified maze in patients with atrial fibrillation undergoing concomitant cardiac surgery. *Journal of Thoracic and Cardiovascular Surgery*.

[2] Haïssaguerre M, Jaïs P, Shah DC (1996). Right and left atrial radiofrequency catheter therapy of paroxysmal atrial fibrillation. *Journal of Cardiovascular Electrophysiology*.

[3] Park HE, Kim KH, Kim KB, Ahn H, Choi YS, Oh S (2010). Characteristics of P wave in patients with sinus rhythm after maze operation. *Journal of Korean Medical Science*.

[4] Legato MJ, Ferrer MI (1974). Intermittent intra-atrial block: its diagnosis, incidence and implications. *Chest*.

[5] Lin YJ, Tai CT, Kao T (2005). Electrophysiological characteristics and catheter ablation in patients with paroxysmal right atrial fibrillation. *Circulation*.

